# Sex-specific differences in nociceptive behaviour and the amygdala endocannabinoid system in the rat monoiodoacetate-induced knee osteoarthritis model

**DOI:** 10.1016/j.ynpai.2026.100227

**Published:** 2026-07-19

**Authors:** Mehnaz I. Ferdousi, Rosmara Infantino, Maria C. Redmond, Santhosh D. Krishnan, Canny K. Adjei, Barry McDermott, Alison Liddy, Leo Quinlan, Martin O'Halloran, David P. Finn

**Affiliations:** a Pharmacology and Therapeutics, School of Pharmacy and Medical Sciences, Institute for Health Discovery and Innovation, University of Galway, Galway City, Ireland; b Physiology, School of Pharmacy and Medical Sciences, University of Galway, Galway City, Ireland; c Galway Neuroscience Centre, University of Galway, Galway City, Ireland; d Centre for Pain Research, University of Galway, Galway City, Ireland; e Translational Medical Device Lab, University of Galway, Galway City, Ireland; f Relevium Medical, Business Innovation Centre, Upper Newcastle, Galway City, Ireland

**Keywords:** Osteoarthritis, Chronic pain, Sex differences, Oestrous cycle, MIA model, Endocannabinoid system, Amygdala

## Abstract

**Introduction:**

Osteoarthritis (OA) is a chronic, progressive joint disorder with higher prevalence and pain severity in women than men. The endocannabinoid system (ECS) modulates pain and shows sexual dimorphism, yet sex-specific alterations in central ECS signalling in OA pain remain under-investigated.

**Objectives:**

We investigated sex-dependent differences in pain-related behaviours and central ECS signalling in the rat monoiodoacetate (MIA) model of knee OA pain.

**Methods:**

Adult male and female Sprague-Dawley rats (8-9 weeks old) received an intra-articular injection of either MIA (2 mg/50 μL) or saline (Sham control) in the left knee. Pain-related behaviours were assessed over 63 days post-injection. The influence of oestrous cycle stage on pain-related behaviours was examined in 100 additional MIA-injected female rats. Endocannabinoids [anandamide (AEA), 2-arachidonoylglycerol (2-AG)] and *N*-acylethanolamines [palmitoylethanolamide (PEA), oleoylethanolamide (OEA)] were quantified in brain and spinal cord regions (LC-MS/MS), and ECS-related gene expression (*Faah*, *Mgll*, *Cnr1*, *Cnr2*) was assessed (RT-qPCR).

**Results:**

MIA females exhibited greater weight-bearing deficits and hind paw mechanical hypersensitivity than males throughout the study period. Oestrous cycle stage did not influence MIA-induced pain-related behaviours. MIA males showed lateralised ECS signalling in the amygdala, with elevated endocannabinoid and *N*-acylethanolamine levels in the ipsilateral/left amygdala, a pattern absent in females. A bilateral reduction of *Mgll* expression in the amygdala was observed in MIA female rats compared to Sham controls.

**Conclusions:**

In the MIA model, female rats demonstrate greater nociceptive behaviour and a different amygdala ECS profile, compared with males, paving the way for further investigation of ECS-mediated mechanisms underlying sex differences in OA pain.

## Highlights

• Female rats show greater nociceptive behaviour than males in the MIA knee OA model.
• Oestrous cycle does not influence OA pain development or severity in MIA females.
• MIA males and females have a different amygdala endocannabinoid system profile.

## Graphical abstract

**Figure:**
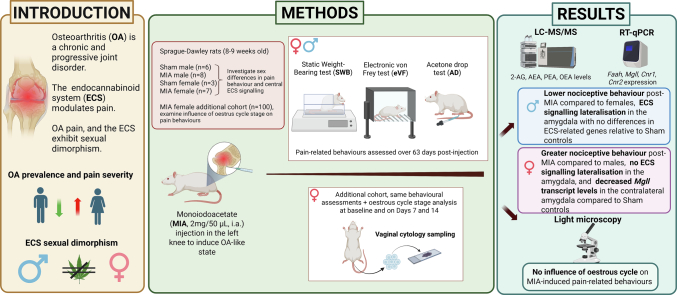

## 1 Introduction

Osteoarthritis (OA) is a disabling condition affecting over 500 million people worldwide (Tang et al., 2025). Defined as a chronic disorder of movable joints, and characterised by cellular stress and extracellular matrix degradation (Kraus et al., 2015), OA is now recognised as a complex and multifactorial condition involving all joint tissues (Tang et al., 2025). Women show a higher prevalence of OA compared to men, and greater OA-related pain severity, especially for knee OA (Di et al., 2024). Anatomical, biomechanical, and hormonal factors have been suggested to account for these sex differences (Segal et al., 2024), alongside the recognised sexual dimorphism in pain sensitivity observed across multiple chronic pain conditions (Bartley and Fillingim, 2013; Fillingim et al., 2009; Finn et al., 2026; Mogil, 2020).

Notably, clinical and preclinical studies demonstrate that females display distinct features compared to males in diverse aspects related to OA pain pathophysiology (Segal et al., 2024), including inflammatory responses (Mun et al., 2020; Perruccio et al., 2019), neuroimmune interactions (Luo et al., 2021; Tavares-Ferreira et al., 2022; Valdrighi et al., 2022), peripheral and central sensitisation mechanisms (Bartley et al., 2016; Chen et al., 2020; Diogenes et al., 2006; Sannajust et al., 2019), and hormonal influence on pain perception (Aloisi and Bonifazi, 2006; Hellström and Anderberg, 2003). Despite this growing evidence of sex differences in OA pain, comprehensive behavioural characterisation of chronic OA-related pain in male and female rodent models remains limited, particularly in long-term experimental settings.

The endocannabinoid system (ECS) plays a pivotal role in pain perception and modulation (Starowicz et al., 2017; Woodhams et al., 2017), and exhibits sexual dimorphism, with physiological, pathological, and pharmacological implications (Blanton et al., 2021; Boullon et al., 2023, 2021; Craft et al., 2013; Henderson-Redmond et al., 2021; Llorente-Berzal et al., 2022). To date, ECS involvement in monoiodoacetate (MIA)-induced OA pain and related depressive behaviour has been demonstrated (Burston et al., 2013; Kędziora et al., 2023; La Porta et al., 2013). However, the role of the ECS in sex-related OA pain differences remains largely unexplored.

Here, we provide a longitudinal behavioural assessment of MIA-induced knee OA pain in male and female rats across an extended chronic phase (up to 63 days post-MIA), and examine the influence of the oestrous cycle on behavioural outcomes in female MIA rats. We further investigated any sex-related alterations of the central ECS in spinal cord and various brain regions involved in pain processing and modulation.

## 2 Methods

Detailed methodology is reported in **Supplementary** Methods and Results.

Briefly, adult Sprague-Dawley rats (*n* = 24, 3-9 per experimental group) were used in the male versus female comparative study. Data from an additional 100 MIA-injected female rats were analysed to assess oestrous cycle influence on knee OA pain-related behaviours, allowing for appropriate statistical power. The sample size was determined based on similar studies reported in the literature (Ro et al., 2020). All procedures were approved by the Animal Care and Research Ethics Committee, University of Galway, and licensed by the Health Products Regulatory Authority, Ireland, in compliance with EU Directive 2010/63. The study is reported in line with the ARRIVE 2.0 Essential 10 guidelines (Percie du Sert et al., 2020). All in vivo work was carried out by female experimenters.

After baseline behavioural assessments, MIA (2 mg/50 μL) was injected intra-articularly into the left knee under anaesthesia to induce an OA-like state (Pomonis et al., 2005). Sham controls received an equivalent volume of saline under similar conditions. Pain-related behaviours – static weight-bearing (SWB) (Bove et al., 2003), mechanical hypersensitivity (electronic von Frey, eVF) (Di Marino et al., 2024), and cold hypersensitivity (acetone drop, AD) (Yoon et al., 1994) – were assessed for 63 days post-injection. On Day 64, rats were euthanised, brains and spinal cords harvested, and the amygdala, periaqueductal grey, rostral ventral medulla, prefrontal cortex, thalamus, and dorsal lumbar spinal cord (L3-L6) were gross-dissected. Endocannabinoid [anandamide (AEA), 2-arachidonoylglycerol (2-AG)] and *N*-acylethanolamine [palmitoylethanolamide (PEA), oleoylethanolamide (OEA)] levels were quantified using LC-MS/MS (Boullon et al., 2021; Corcoran et al., 2020; Kerr et al., 2012), and ECS-related gene expression [fatty acid amide hydrolase (*Faah)*, monoacylglycerol lipase (*Mgll)*, cannabinoid receptor type 1 (*Cnr1)*, and cannabinoid receptor type 2 (*Cnr2*)] was assessed using RT-qPCR (Di Marino et al., 2024), as described previously.

The additional female cohort (*n* = 100) underwent the same MIA induction and then behavioural assessments at baseline and on Days 7 and 14 only. Oestrous stage was determined by vaginal cytology (McLean et al., 2012). Representative images of the different stages of the oestrous cycle are shown in **Suppl. Fig. 1**.

Data are presented as mean ± SEM. Normality and homogeneity of variance were assessed using the Shapiro-Wilk and Levene's tests, respectively. Outliers were identified using the ROUT method (Q = 1%) (Motulsky and Brown, 2006), and excluded from biochemical analyses only. No behavioural data were excluded. Behavioural time-course data were analysed using three-way repeated measures (RM) ANOVA (within-subjects factor: time, between-subjects factors: sex and model). Neurochemical and gene expression data were analysed using two-way (factors: sex and model) or three-way ANOVA (factors: sex, model, and side), or the corresponding mixed-effects model when missing values resulted from outlier removal. Tukey's post hoc test was used for multiple comparisons. Unpaired *t*-tests with Welch's correction, Fisher's exact test, Wilcoxon one-sample *t*-test, or binomial logistic regression were applied, where appropriate (see **Suppl. Material**). Statistical significance was set at *p* < 0.05. Analyses were performed using GraphPad Prism 10.6.1.

## 3 Results

### 3.1 Female rats display greater MIA-induced nociceptive behaviours than male rats

A time-course analysis of pain-related behaviours was conducted in male and female MIA rats to characterise any sex differences.

**Fig. 1:**
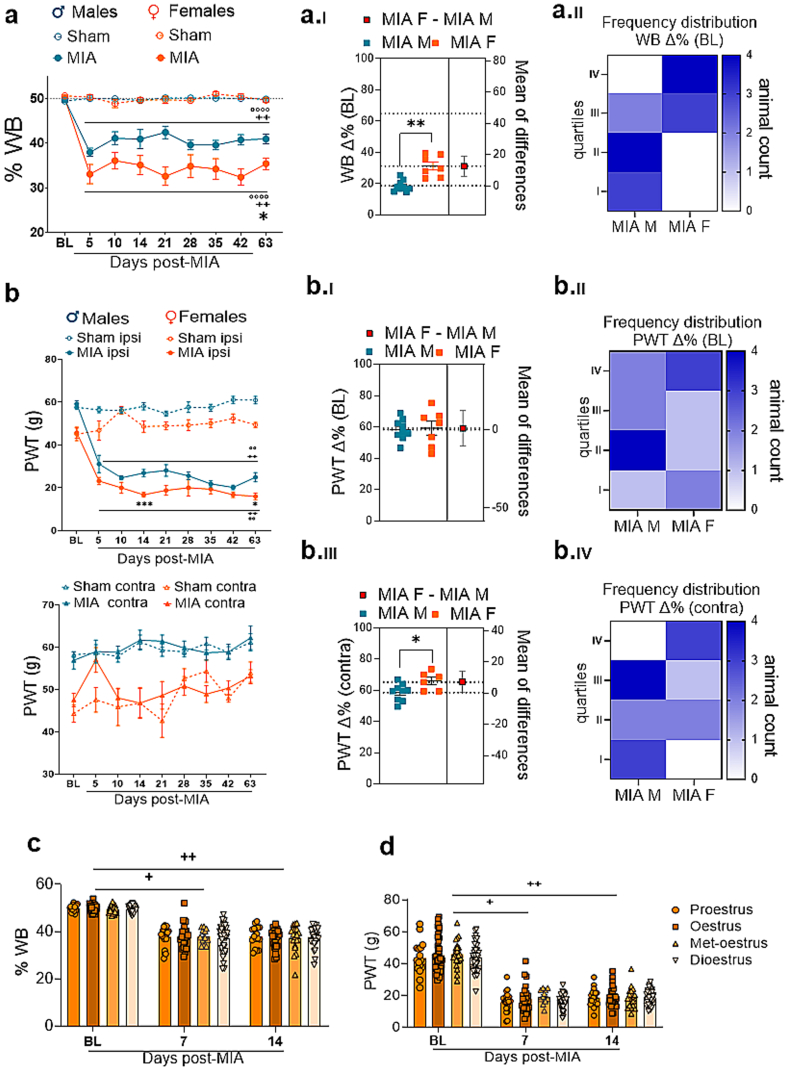
MIA-induced knee OA pain-related behaviours in male and female rats and across oestrous cycle stages in female rats. (a) Static weight-bearing test (%WB on ipsilateral/left hind limb) in male and female MIA or Sham rats; (a.I) Estimation plot of WB∆% relative to baseline (BL) in MIA rats; (a.II) Heatmap of WB∆%(BL) frequency distribution in males vs females MIA rats. (b) Electronic von Frey test (PWT, g) on ipsilateral (upper panel) and contralateral (lower panel) hind paws in male and female MIA or Sham rats; (b.I) Estimation plot of ipsilateral PWT∆% relative to BL in MIA rats; (b.II) Heatmap of PWT∆%(BL) frequency in males vs females MIA rats; (b.III) Estimation plot of ipsilateral PWT∆% relative to contralateral hind paw (contra) in MIA rats; (b.IV) Heatmap of ipsilateral PWT∆%(contra) frequency in male vs female MIA rats. Data are expressed as mean ± SEM (MIA: *n* = 8 males, *n* = 7 females; Sham: *n* = 6 males, *n* = 3 females). Time-course data were analysed by three-way repeated measures ANOVA with Tukey's (a and b) post hoc test. Mean differences (a.I, b.I, b.III) were analysed by unpaired *t*-test with Welch's correction. (c and d) Behavioural responses of female MIA rats across the four oestrous cycle stages in static weight-bearing test (ipsilateral %WB) (c) and electronic von Frey test (PWT, g) (d) at baseline, Day 7, and Day 14 post-injection. Data are expressed as mean ± SEM (*n* = 100) and analysed using two-way ANOVA followed by Tukey's post hoc test (c-d). Symbols: °°*p* < 0.01, °°°°*p* < 0.0001 vs respective Sham controls; *p < 0.05, **p < 0.01 vs MIA males; ++p < 0.01 vs BL. Abbreviation: M = males, F = females.

In the SWB test, three-way RM ANOVA revealed significant main effects of time (F_(8,191)_ = 11.17, *p* < 0.0001), sex (F_(1,24)_ = 100.80, *p* = 0.001), and model (F_(1,24)_ = 235.00, p < 0.0001) with significant interaction effects of time x model (F_(8, 191)_ = 11.42, p < 0.0001), and sex x model (F_(1,24)_ = 14.48, p = 0.001) on ipsilateral hind limb weight-bearing deficits, expressed relative to the contralateral hind limb (%WB). From Day 5 onward, MIA-injected animals of both sexes displayed persistent ipsilateral %WB reduction compared with baseline (*p* < 0.01) and compared with Sham controls (p < 0.0001) (Fig. 1a). MIA Females consistently exhibited greater deficits than MIA males throughout the observation period, with the largest differences observed on Day 21 (Day 21: 32.62 ± 2.06% vs 42.42 ± 1.40%, *p* < 0.02; Fig. 1a). In the eVF test, ipsilateral and contralateral PWTs were analysed separately. Analysis of ipsilateral PWTs revealed significant main effects of time (F_(5.535, 132.8)_ = 19.10, p < 0.0001), sex (F_(1,24)_ = 36.24, p < 0.0001), and model (F_(1,24)_ = 482.60, p < 0.0001), with significant interaction effects of time x sex (F_(5.535, 132.8)_ = 2.31, p < 0.0001) and time x model (F_(5.535, 132.8)_ = 31.36, p < 0.0001). Both sexes developed long-lasting MIA-induced mechanical hypersensitivity of the ipsilateral hind paw from Day 5, with reduced PWT versus baseline (p < 0.01) and versus Sham controls (p < 0.01) (Fig. 1b). MIA females showed greater ipsilateral hind paw mechanical hypersensitivity than MIA males at both early and late time points throughout the observation period (Day 14: 16.73 ± 1.11 g vs 26.92 ± 1.62 g, *p* = 0.0008; Day 63: 15.99 ± 1.54 g vs 24.93 ± 2.17 g, *p* = 0.022; Fig. 1b). No differences were observed in contralateral PWTs or in male or female Sham controls relative to baseline. Percentage change indices (Δ%) for post-MIA nociceptive behaviours were therefore calculated only in MIA rats, either relative to baseline (BL) or by comparing the ipsilateral with the contralateral side. Females showed significantly higher WB%∆(BL) than males (31.21 ± 2.55% vs 18.55 ± 1.19%; *t* = 4.49, df = 8.57, *p* = 0.002; unpaired *t*-test with Welch's correction; Fig. 1a**.I**) and larger PWT%∆(contra), (66.02 ± 2.36% vs 58.65 ± 1.83%; *t* = 2.47, df = 10.45, *p* = 0.032; unpaired t-test with Welch's correction; Fig.1b**.III**), whereas PWT%∆(BL) did not differ between sexes (Fig. 1b**.I**). Frequency distribution analysis of these indices (Fig. 1a**.II, 1b.II, 1b.IV**) revealed a significant difference in WB%∆ relative to BL (Fisher's exact test, *p* = 0.004; Fig. 1a**.II**), and eVF%∆ relative to contralateral side (Fisher's exact test, *p* = 0.024; Fig. 1b**.IV**), with most males distributed in lower quartiles (I-II) and females in the upper quartiles (III-IV). No sex-related differences were observed in cold hypersensitivity (AD test, **Suppl. Fig. 2**).

### 3.2 The oestrous cycle did not influence knee OA-related pain phenotype in female MIA rats

Given the observed sex differences in MIA-induced pain-related behaviours, we investigated whether oestrous cycling could account for variability in female responses. The influence of oestrous stage on ipsilateral %WB and PWT was assessed in a separate cohort of 100 female MIA rats at baseline and on Days 7 and 14 post-MIA (Fig.1c and d). Two-way ANOVA revealed a significant effect of time on both %WB (F_(11, 318)_ = 77.49, *p* < 0.0001) (Fig.1c) and PWT (F_(11, 318)_ = 90.18, *p* < 0.0001) (Fig.1d), but no significant effect of oestrous stage or time x oestrous stage interaction was found. Binomial logistic regression confirmed that the oestrous cycle stage did not predict responder status for %WB or PWT at any of the three timepoints (all *p* > 0.05; **Suppl. Tables 1 and 2**).

### 3.3 MIA rats exhibit sex-dependent differences in endocannabinoid levels and *Mgll* expression in the amygdala

To explore neurobiological mechanisms underlying sex differences, we next examined ECS signalling in key pain-related brain regions and dorsal lumbar spinal cord.

**Fig. 2:**
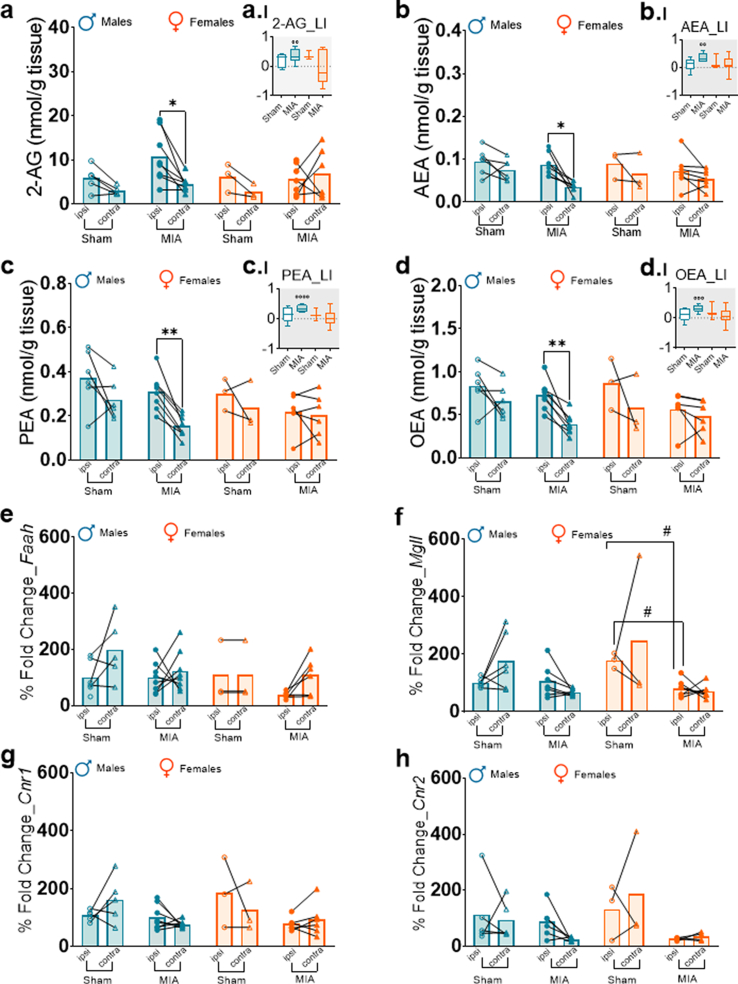
Levels of endocannabinoids and *N*-acylethanolamines and expression of ECS-related genes in the ipsilateral (left) and contralateral (right) amygdala of male and female MIA or Sham rats. (a–d) Endocannabinoid (2-AG, AEA) and *N*-acylethanolamine (PEA, OEA) levels in the left and right amygdala of male and female MIA or Sham rats; (a.I-d.I) Lateralisation index (LI) of respective analytes. (e–h) Expression of *Faah*, *Mgll*, *Cnr1*, and *Cnr2* transcripts in the left and right amygdala of male and female MIA or Sham rats as a percentage of levels in Sham male ipsi/left samples. Data are expressed as mean ± SEM (n = 3-9 per group) and analysed using three-way ANOVA (or corresponding mixed effects model due to outlier removal) with Tukey's post hoc test (a-h) or Wilcoxon one-sample t-test (a.I-d.I). Symbols: *p < 0.05, **p < 0.01 vs contra/right amygdala; °°p < 0.01, °°°*p* < 0.001, °°°°p < 0.0001 vs LI = 0; #p < 0.05 vs Sham control. Abbreviation: M = males, F = females.

Analysis of endocannabinoid and *N*-acylethanolamine levels in the left versus right amygdala revealed sex-dependent lateralisation. MIA males consistently showed higher ipsilateral/left amygdala levels than contralateral/right side across all analytes: 2-AG (side: F_(1,26)_ = 4.68, *p* = 0.04; 10.66 ± 2.05 nmol/g tissue vs 4.50 ± 0.63 nmol/g tissue, *p* = 0.046; Fig. 2a), AEA (side: F_(1,26)_ = 9.08, *p* = 0.005, model: F_(1,14)_ = 5.71, *p* = 0.03; 0.088 ± 0.009 nmol/g tissue vs 0.035 ± 0.005 nmol/g tissue, *p* = 0.006; Fig. 2b), PEA (side: F_(1,26)_ = 7.36, p = 0.006; model: F_(1,12)_ = 10.48, *p* = 0.007; 0.31 ± 0.03 nmol/g tissue vs 0.15 ± 0.03 nmol/g tissue, p = 0.006; Fig. 2c), and OEA (side: F_(1,26)_ = 10.42, *p* = 0.003; model: F_(1,12)_ = 11.46, p = 0.005; 0.73 ± 0.06 nmol/g tissue vs 0.39 ± 0.043 nmol/g tissue, *p* = 0.008; Fig. 2d). This lateralisation pattern in the analytes was absent in the MIA females, and in Sham controls of both sexes (Fig. 2a-d). Lateralisation indices (LI) were calculated for all analytes (Fig. 2a**.I-d.I**), as described in **Suppl. Methods**, and analysed with Wilcoxon one-sample *t*-test against a theoretical LI = 0 (i.e. no lateralisation). Lateralisation was significant for all analytes in MIA males (2-AG: *t* = 4.43, df = 7, *p* = 0.0034; AEA: *t* = 5.58, df = 6, *p* = 0.0014; PEA: *t* = 8.19, df = 7, p < 0.0001; OEA: *t* = 7.13, df = 7, *p* = 0.0002). Endocannabinoid and *N*-acylethanolamine levels in other regions did not differ between sexes (**Suppl. Fig. 3**).

Finally, gene expression analysis in the amygdala revealed that *Faah*, *Cnr1*, and *Cnr2* transcripts showed no lateralisation and no sex differences (Fig. 2e, g and h). In contrast, there was a significant effect of model on *Mgll* expression (F_(1,39)_ = 11.70, *p* = 0.0015; Fig. 2f), with lower levels in MIA females than Sham controls in contralateral/right (70.12 ± 8.98% vs 245.82 ± 149.21%, *p* = 0.036) amygdala, consistent with a trend toward higher 2-AG levels in contralateral/right amygdala of MIA females compared to Sham females.

## 4 Discussion

The present study provides evidence for sex differences in pain-related behaviours in the MIA-induced knee OA rat model. Previous studies reported sex- and age-dependent differences over shorter time frames (up to 21 days post-MIA), showing that aged female rats display greater weight-bearing deficits and hind paw mechanical hypersensitivity (Ro et al., 2020). Our findings extend this observation demonstrating that sex differences in OA-related pain phenotype persist long-term up to 63 days post-MIA. Specifically, female MIA rats showed lower ipsilateral %WB, suggestive of greater spontaneous pain-related behaviour at the knee OA-like lesion site. This finding aligns with clinical observations that women with knee OA report more severe pain than men (Segal et al., 2024). Additionally, female MIA rats displayed greater hind paw mechanical hypersensitivity than males. This observation is consistent with reports that female rodents are more prone to central sensitisation and developing secondary hypersensitivity, not only in MIA-induced knee OA (Ro et al., 2020), but also in other MIA-induced arthropathy models, including a temporomandibular joint disorder model (Sannajust et al., 2019).

Notably, the oestrous cycle stage did not affect MIA-induced pain-related behaviours. Although higher oestrogen levels have been associated with reduced pain sensitivity in chronic pain conditions (Aloisi and Bonifazi, 2006; Hellström and Anderberg, 2003) and protection of joint structures in humans (Boyan et al., 2013; Jin et al., 2017; Roman-Blas et al., 2009; Welhaven et al., 2024) and animal models (Calvo et al., 2007; Høegh-Andersen et al., 2004; Roman-Blas et al., 2009; Sniekers et al., 2008; Yang et al., 2012), findings in OA remain equivocal (Cirillo et al., 2006; Segal et al., 2024). In our study, oestrous stage at the time of MIA injection did not influence the development of the OA-like pain phenotype, suggesting that physiological higher oestrogen levels in oestrus and proestrus phases are not sufficient to exert protective effects. Nonetheless, dose-dependent effects of oestrogen replacement therapy have been described (Sahyoun et al., 1999). Moreover, there is well-documented dissociation between structural joint damage and pain severity (Bedson and Croft, 2008; Dieppe and Lohmander, 2005; Fukui et al., 2010; Jin et al., 2017), suggesting additional neurobiological mechanisms that could modulate pain in a sex-dependent manner (Bartley and Fillingim, 2013; Mogil, 2020; Segal et al., 2024), and reinforcing the necessity of adopting sex-inclusive approaches in both clinical and preclinical research (Finn et al., 2026).

In this context, the present study identified, for the first time, sex-specific alterations in the MIA model for the ECS within the amygdala, a key region involved in both sensory and affective dimensions of pain processing (Finn et al., 2003; Neugebauer, 2015; Thompson and Neugebauer, 2017). The ECS is sexually dimorphic and plays a critical role in central sensitisation and maladaptive plasticity in chronic pain (Finn et al., 2021; Woodhams et al., 2017). While CB_2_ receptor involvement in spinal sensitisation has been reported in male MIA rats (Burston et al., 2013; La Porta et al., 2013), and CB_1_ receptors have been implicated in MIA-induced knee OA pain and associated depressive-like behaviours in male rats (Kędziora et al., 2023), sex-specific ECS alterations in supraspinal regions remain under explored. Our neurochemical analyses revealed lateralised amygdala ECS signalling in MIA males, a pattern not seen in MIA females. Previous studies in other rat chronic pain models, including spared nerve injury (Boullon et al., 2021) and chemotherapy-induced peripheral neuropathy (Di Marino et al., 2024), found no sex-specific differences in amygdala endocannabinoid content under either pathological or control/sham conditions. This study reveals, for the first time, a sex-specific divergence in this pattern in the MIA model.

Amygdala lateralisation in pain processing is well-recognised (Neugebauer, 2015), with the right central amygdala (CeA) associated with maladaptive pronociceptive plasticity (Allen et al., 2021; Ji and Neugebauer, 2009; Neugebauer, 2015), whereas the left CeA may serve protective, non-pronociceptive functions (Allen et al., 2021; Sadler et al., 2017). In models of OA and formalin-induced persistent inflammatory pain, male rodents showed central sensitisation within the right CeA, dependent on protein kinase A (PKA) (Bird et al., 2005; Fu et al., 2008; Han et al., 2005) and extracellular signal-regulated kinase (ERK) activation (Carrasquillo and Gereau 4th, 2007, Carrasquillo and Gereau 4th, 2008), which was not observed in the left amygdala (Ji and Neugebauer, 2009). Interestingly, PKA and ERK are modulated by CB_1_ and CB_2_ downstream signalling (Han et al., 2022; Howlett et al., 2002; Leo and Abood, 2021), influencing neural transmission patterns. Our finding of elevated 2-AG and AEA in the left/ipsilateral amygdala of MIA males, but not females, suggests a possible sex-dependent role for ECS signalling in hemispheric lateralisation related to OA pain. The concomitant increase in PEA and OEA levels in the left/ipsilateral amygdala of MIA males further suggest potential sex-specific ECS-mediated anti-inflammatory mechanisms. Both PEA and OEA exert anti-inflammatory effects via PPAR-α activation (Guida et al., 2017; Li et al., 2023; Luo et al., 2019; Pérez-Martín et al., 2023; Re et al., 2007) and can compete for endocannabinoid-degrading enzymes, thereby sustaining elevated endocannabinoid levels (Iannotti et al., 2016; Iannotti and Vitale, 2021), consistent with our findings. The present study also revealed a bilateral reduction in *Mgll* expression in the amygdala of MIA females compared to Sham controls. This sex-specific alteration in amygdala *Mgll* transcript levels appears to be distinct from that reported in other chronic pain models, such as the hind limb ischemia-reperfusion injury model (Healy et al., 2026), suggesting that OA pain may engage the ECS in a manner that differs from other persistent pain states. Collectively, these findings further highlight sex-dependent adaptations of the amygdala ECS in chronic OA pain.

Altogether, these findings demonstrate that female rats, independent of oestrous cycle stage, exhibit greater nociceptive behaviours than males in the MIA model of knee OA pain. We identify, for the first time, sex-specific patterns in the amygdala ECS signalling in this model, with MIA males showing left-sided lateralisation of ECS tone that is absent in female counterparts. Although the functional significance of this lateralisation remains to be established, these findings suggest that distinct neurobiological mechanisms may underlie sex differences in OA pain. This initial evidence thus calls for further investigation, potentially opening new paths toward mechanism-based and sex-informed personalised therapeutic approaches for OA pain.

## CRediT authorship contribution statement

**Mehnaz I. Ferdousi:** Writing – review & editing, Methodology, Investigation, Formal analysis, Data curation, Conceptualization. **Rosmara Infantino:** Writing – original draft, Methodology, Investigation, Formal analysis, Data curation, Conceptualization. **Maria C. Redmond:** Writing – review & editing, Methodology, Investigation, Formal analysis. **Santhosh D. Krishnan:** Writing – review & editing, Methodology, Investigation, Formal analysis. **Canny K. Adjei:** Writing – review & editing, Methodology, Investigation, Formal analysis. **Barry McDermott:** Writing – review & editing, Methodology, Conceptualization. **Alison Liddy:** Writing – review & editing, Supervision, Funding acquisition. **Leo Quinlan:** Writing – review & editing, Supervision, Funding acquisition. **Martin O'Halloran:** Writing – review & editing, Supervision, Funding acquisition. **David P. Finn:** Writing – review & editing, Supervision, Resources, Project administration, Conceptualization.

## Declaration of competing interest

The authors declare the following financial interests/personal relationships which may be considered as potential competing interests: Martin O'Halloran, Alison Liddy, Leo Quinlan, David Finn reports financial support was provided by European Innovation Council (EIC). Martin O'Halloran reports financial support was provided by European Research Council (ERC). Alison Liddy and Mehnaz Ferdousi reports a relationship with Relevium Medical that includes: employment. Martin O'Halloran, Alison Liddy, David Finn reports a relationship with Relevium Medical that includes: equity or stocks. If there are other authors, they declare that they have no known competing financial interests or personal relationships that could have appeared to influence the work reported in this paper.

## Data Availability

Data will be made available on request.

## Appendix Supplementary Methods and Results

Supplementary Methods and Results
