# Supplementary material for: Sex-specific differences in nociceptive behaviour and the amygdala endocannabinoid system in the rat monoiodoacetate-induced knee osteoarthritis model
Source: Neurobiol Pain. 2026 Jul 19;20:100227. doi: 10.1016/j.ynpai.2026.100227 (PMC13430258; doi:10.1016/j.ynpai.2026.100227)
Supplement: Supplementary file 1 — Supplementary Methods and Results [file mmc1.docx]

**Supplementary Methods**

*Animals*

A total of 24 adult Sprague-Dawley rats (MIA: n=8 males, n=7 females; Sham: n=6 males, n=3 females; 8-9 weeks old on arrival, Charles River UK) were used for this study. Data from an additional cohort of 100 MIA-injected female rats (8-9 weeks on arrival, Charles River UK), obtained from ongoing studies in our laboratory using the same model, were analysed to evaluate the influence of oestrous cycle on pain-related behaviours. The sample size was determined based on similar studies reported in the literature (Ro et al., 2020). Rats were housed 2-3 per cage under standard conditions (22±2 °C, 55-65% humidity, 12/12 h light/dark cycle, lights on at 07:00 h). Food and water were available *ad libitum,* and environmental enrichment (plastic tube and nesting material Sizzlenest®) was provided in the home cages.

The experimental procedures were approved by the Animal Care and Research Ethics Committee (ACREC), University of Galway, and performed under license from the Health Products Regulatory Authority in the Republic of Ireland (AE19125/P104), and in accordance with EU Directive 2010/63. The study is reported in line with the ARRIVE 2.0 Essential 10 guidelines (Percie du Sert et al., 2020). All *in vivo* work was carried out by female experimenters. Each rat was considered to be an experimental unit.

*MIA-induced knee OA*

An OA-like state was induced by administering a single intra-articular (i.a.) injection of MIA (2 mg/50 µL) into the left knee joint (Pomonis et al., 2005), using a sterile 30G needle-tipped insulin syringe at a volume of 50 µL under brief anaesthesia (2-3% isoflurane, 0.8 L/min O_2_). Sham animals received an equivalent volume of sterile saline (NaCl 0.9%) under similar conditions. The needle was inserted perpendicularly through the infrapatellar ligament into the joint space of the left tibiofemoral joint. The animals were returned to the home cage after anaesthetic recovery.

*Experimental timeline*

Upon arrival, animals were acclimated for one week. Two baseline (BL) measurements in the static weight-bearing, electronic von Frey, and acetone drop tests were obtained prior to MIA induction, averaged, and reported as BL. On Day 0, rats received an i.a.injection of either MIA or saline in the left knee and were allowed to recover. Pain-related behaviours (static weight-bearing, electronic von Frey, and acetone drop tests) were further assessed on Days 5, 10, 14, 21, 28, 35, 42, and 63 post-MIA injection. On Day 64, animals were euthanised by decapitation, and various brain regions and dorsal lumbar spinal cords were dissected, snap-frozen on dry ice, and stored at -80°C until further neurochemical and biomolecular analyses.

An additional cohort of 100 female rats underwent the same acclimation, BL assessment, and MIA injection. In contrast to the main timeline, these animals were tested at two critical time points: Day 7 (MIA model inflammatory phase) and Day 14 (MIA model chronic phase establishment). After completing behavioural testing at each of the 3 time points (BL, Day 7 and Day 14), vaginal swabbing was performed to determine oestrous cycle stage according to previously described criteria (McLean et al., 2012).

Representative images of the different oestrous stages are shown in **Suppl. Fig. 1**. Thereafter, animals continued in separate experimental protocols not reported here. This cohort provided sufficient statistical power to evaluate the potential influence of oestrous cycle stage on pain-related behaviours.

*Static weight bearing test*

Spontaneous knee joint pain-related behaviour (primary site) was assessed with the static weight-bearing test (Bove et al., 2003), using the incapacitance tester (Linton Instrumentation, UK). Animals were habituated to the apparatus for two days prior to baseline assessment (5 min each day). For testing, rats were placed with forepaws on the angled surface and hind paws on force sensor plates. The force (g) exerted by each hind paw was averaged over 3 s. Percentage weight-bearing (%WB) on the ipsilateral (left) hind limb was calculated using the following equation. Three readings per rat were taken, and mean %WB calculated.

$$\%WB=\frac{Weight on ipsilateral hind limb}{Weight on contralateral+ipsilateral hind limbs} \times100$$

The weight-bearing percentage variation relative to baseline [WB%∆(BL)], for each measurement between Day 5 and Day 63, was calculated as:

$$WB\%\Delta(BL)=\frac{\%WB on ipsilateral hind limb\left( Day 5-63 \right)-\%WB on ipsilateral hind limb (BL)}{\%WB on ipsilateral hind limb (BL)} \times100$$

Values were then averaged to obtain a single representative index for each animal.

*Electronic von Frey test*

Mechanical hypersensitivity at the ipsilateral hind paw (secondary site) compared to the healthy contralateral side was measured using an electronic von Frey (eVF) (Di Marino et al., 2024) aesthesiometer (IITC Life Science, USA). After 20 min acclimatisation on a mesh floor, gradual pressure was applied with a rigid probe tip to the plantar surface of each hind paw. Paw withdrawal threshold (PWT) was recorded as the force (g) eliciting paw withdrawal, flinching, or licking. Each paw was tested three times (≥5 min interval), starting with the contralateral side, and average PWT calculated.

The PWT percentage variation relative to baseline [PWT%∆(BL)], or relative to the contralateral hind paw [PWT%∆(contra)], for each measurement between Day 5 and Day 63, was calculated as:

$$PWT\%\Delta(BL)=\frac{PWT ipsilateral hind limb\left( Day 5-63 \right)-PWT ipsilateral hind limb (BL)}{PWT ipsilateral hind limb (BL)} \times100$$

$$PWT\%\Delta(contra)=\frac{PWT ipsilateral hind limb\left( Day 5-63 \right)-PWT contralateral hind limb}{PWT ipsilateral hind limb (Day 5-63)} \times100$$

Values were then averaged to obtain a single representative index for each animal.

*Acetone drop test*

Cold hypersensitivity of the ipsilateral hind paw (secondary site) compared to the healthy contralateral side was assessed using acetone drop test, performed 15 min after the von Frey test and in the same apparatus, as previously described (Yoon et al., 1994). Starting with the contralateral side, a drop of acetone was applied to the central surface of the hind paw using a 1 mL syringe. The latency (s) to the first response (paw withdrawal, flinching, licking, or shaking) following acetone application and the total number of responses within 60 seconds were recorded. The test was repeated three times and measurements averaged for each side per rat.

*LC-MS/MS measurement of endocannabinoids and* *N-acylethanolamines*

Endocannabinoid (AEA, 2-AG) and related *N*-acylethanolamine (PEA, OEA) levels were measured in key regions involved in pain: amygdala, periaqueductal grey, rostral ventral medulla, prefrontal cortex, thalamus, and dorsal lumbar (L3-L6) spinal cord, following established protocols (Boullon et al., 2021; Di Marino et al., 2024; Redmond et al., 2025). Samples were analysed using an Agilent 1260 Infinity II HPLC system coupled to a Sciex QTRAP 4500 MS. Each sample (5 μL) was injected from a cooled autosampler (4 °C) onto an Agilent Zorbax column (50 cm × 2.1 cm). Analytes were separated based on polarity, with less polar compounds eluting first, and detected by electrospray ionisation. The mobile phase consisted of solution A (HPLC-grade water with 0.1% formic acid) and solution B (100% acetonitrile with 0.1% formic acid) at a flow rate of 0.2 mL.min^-1^. A reverse-phase gradient was applied: 45% B for 1 min, linearly increased to 100% B over 4 min, held at 100% B until 12 min, then returned to initial conditions with 5 min re-equilibration before the next injection. Retention times for 2-AG, AEA, OEA, and PEA were 8.2, 7.9, 8.7, and 8.4 min, respectively. Quantification was performed in MRM mode using deuterated and non-deuterated standards, and data were analysed with Skyline software (v22.2). The lateralisation index (LI) for left (L) and right (R) amygdala analyte levels (nmol/g tissue) was calculated for each animal as LI = (L − R) / (L + R).

*Real time qPCR analysis of endocannabinoid system-related genes*

Real time qPCR analysis was conducted as previously described (Di Marino et al., 2024; Redmond et al., 2025). Total RNA was extracted from the pellets remaining after LC-MS/MS preparation using the NucleoSpin RNA Mini Kit (Macherey-Nagel; Fisher Scientific, Ireland), following the manufacturer’s instructions. RNA concentration, integrity, and purity were determined with a Nanodrop spectrophotometer (ND-1000; Nanodrop, Labtech International, UK). Purity and integrity of samples were evaluated by the absorbance ratios at 260/280 (acceptable values ~2.0) and 260/230 (acceptable values 2.0-2.2), respectively. Samples were normalised to a concentration of 88 ng/μL.

cDNA synthesis was performed using the High-Capacity cDNA Reverse Transcription Kit (ThermoFisher Scientific, Ireland). Gene expression analysis was carried out on an Applied Biosystems StepOne Plus™ instrument (Bio-Sciences, Dun Laoghaire, Ireland).  TaqMan gene expression assays (Biosciences, Ireland) containing forward and reverse primers and FAM-labelled TaqMan probes (Biosciences, Ireland) were used to quantify the genes of interest (*Cnr1* for CB1, assay ID: Rn00562880_m1; *Cnr2* for CB2, assay ID: Rn03993699_s1; *Faah* for FAAH, assay ID: Rn00577086_m1; and *Mgll* for MGL, assay ID: Rn00593297_m1). A VIC-labelled probe for *Actb* (β-actin, assay ID: Rn00667869_m1) was used as the housekeeping control gene. The percentage fold change in gene expression was calculated using the 2^(-ΔΔCt) method, where ΔCt represents the difference between the target gene and the housekeeping gene Ct values, and ΔΔCt represents the difference between the ΔCt of the experimental sample and the ΔCt of the control sample. Results were expressed as a percentage of levels in Sham male ipsilateral (left) samples.

*Oestrous cycle stage analysis*

To assess the oestrous cycle stage in female rats, vaginal smears were collected after behavioural testing at baseline (BL), on Day 7 (inflammatory phase of the MIA model) and Day 14 (establishment of chronic phase of the MIA model) post-injection using sterile, saline-moistened cotton buds. Samples were methanol-fixed, stained with eosin and methylene blue/azure, and examined by light microscopy. Based on established criteria (McLean et al., 2012), the oestrous cycle stage was classified into: (i) proestrus (predominantly characterised by nucleated epithelial cells, **Suppl. Fig. 1a**), (ii) oestrus (cornified squamous epithelial cells in clusters, **Suppl. Fig. 1b**), (iii) met-oestrus (cornified squamous cells and leucocytes, **Suppl. Fig. 1c**), or (iv) dioestrus (mixed nucleated and squamous epithelial cells with numerous leucocytes, **Suppl. Fig. 1d**). For the purpose of analysis, female animals were further divided into two groups based on the oestrogen status on the day of behavioural test: (a) high oestrogen status (proestrus, oestrus) and (b) low oestrogen status (met-oestrus, dioestrus).

**Supplementary Figure 1**
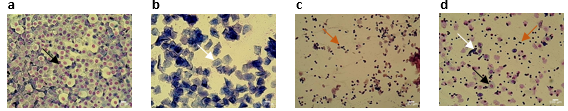


**Suppl. Figure 1.** **Representative images of vaginal cytology samples**. (**a**) Proestrus, (**b**) Oestrus, (**c**) Met-oestrus, and (**d**) Dioestrus stages of the oestrous cycle. Black arrows show nucleated epithelial cells, white arrows show cornified squamous epithelial cells in clusters, and orange arrows show leucocytes.

*Statistics*

Data are presented as mean ± SEM of 3-9 animals per group for behavioural, neurochemical, and biomolecular analyses, and 100 animals for the analysis of the influence of oestrous cycle on pain-related behaviours. Normality and homogeneity were assessed with Shapiro-Wilk and Levene’s tests, respectively. Outliers were identified by the ROUT method (Motulsky and Brown, 2006) with Q=1% (maximum desired false discovery rate) and excluded from analysis. No data points were excluded from the behavioural analyses. However, outliers were identified in the biochemical analyses: one MIA female and one Sham male outlier were identified in the 2-AG dataset, and one MIA female outlier was identified in the PEA and OEA datasets. Additionally, one MIA female outlier was found for *Faah*, one MIA female and one Sham male outlier were identified for *Cnr1*, one MIA male outlier was identified for *Mgll*, and two MIA male and two MIA female outliers were identified for *Cnr2*. Whenever an outlier was identified, both ipsilateral and contralateral values for that animal were excluded.

Time-course behavioural data were analysed using three-way repeated measures (RM) ANOVA (within-subjects factor: time, between-subjects factors: sex and model) followed by Tukey’s post hoc test, where appropriate. Differences between two groups were analysed using unpaired t-tests with Welch’s correction. Differences between frequency distributions were analysed with Fisher’s exact test. Neurochemical levels and gene expression data were analysed using two-way (factors: sex and model) or three-way ANOVA (factors: sex, model, and side). When missing values were present following outlier removal, the corresponding mixed-effects model was used instead. Tukey's post hoc test was used for multiple comparisons, where appropriate. LI of endocannabinoids and *N*-acylethanolamines for each experimental group were tested against a theoretical value of 0 (i.e., absence of lateralisation) using a Wilcoxon one-sample t test. Binomial logistic regression was used to assess whether oestrous cycle stage could predict responder status in pain-related behavioural tests at BL and on Days 7 and 14 post-MIA injection. Binary codes were assigned to both independent and dependent variables. Ipsilateral PWT and %WB were considered dependent variables, with animals classified as high- or low-responders relative to the median value of PWT or %WB. Oestrous cycle stage served as the independent variable, with proestrus/oestrus as high oestrogen status and met-oestrus/dioestrus as low oestrogen status. P<0.05 was considered statistically significant in all cases. Statistical analyses and graphical representations were performed using GraphPad Prism version 10.6.1 (Boston, MA, USA).

**Supplementary Results**

**Supplementary Table 1. Contingency table showing the distribution of low and high responders in static weight-bearing test (%WB) across oestrogen status.** Data were analysed using binomial logistic regression to assess whether oestrous cycle stage could predict responder status in the static weight-bearing test at baseline (BL) and on Days 7 and 14 post-MIA injection. Met-oestrus/dioestrus stages (low oestrogen status), proestrus/oestrus stages (high oestrogen status). Values represent number of animals. Low responder: %WB value below median; high responder: %WB value above median on the test day.

| **Weight-bearing test**  **(% WB)** | **Low responders** | | | **High responders** | | |
| --- | --- | --- | --- | --- | --- | --- |
|  | **BL** | **Day 7** | **Day 14** | **BL** | **Day 7** | **Day 14** |
| Low oestrogen status  (Met-oestrus/dioestrus) | 24 | 18 | 22 | 20 | 14 | 21 |
| High oestrogen status (Proestrus/oestrus) | 26 | 31 | 25 | 30 | 37 | 32 |

**Supplementary Table 2. Contingency table showing the distribution of low and high responders in electronic von Frey test (PWT) across oestrogen status.** Data were analysed using binomial logistic regression to assess whether oestrous cycle stage could predict responder status in von Frey test at baseline (BL) and on Days 7 and 14 post-MIA injection. Met-oestrus/dioestrus stages (low oestrogen status), proestrus/oestrus stages (high oestrogen status). Values represent number of animals. Low responder: PWT value below median, high responder: PWT value above median on the test day.

| **Electronic von Frey test (PWT)** | **Low responders** | | | **High responders** | | |
| --- | --- | --- | --- | --- | --- | --- |
|  | **BL** | **Day 7** | **Day 14** | **BL** | **Day 7** | **Day 14** |
| Low oestrogen status  (Met-oestrus/dioestrus) | 17 | 15 | 19 | 27 | 17 | 24 |
| High oestrogen status (Proestrus/oestrus) | 33 | 37 | 28 | 23 | 31 | 29 |

**Supplementary Figure 2**

**Suppl. Figure 2.** **Cold hypersensitivity in male and female MIA or Sham rats assessed using acetone drop test.** (**a, b**) Latency to respond (s) and (**c,d**) total number of responses in 60 s to acetone drop application on ipsilateral **(a,c)** and contralateral hind paws **(b,d)**. Data are expressed as mean ± SEM (MIA: n=8 males, n=7 females; Sham: n=6 males, n=3 females), and analysed using three-way repeated measures ANOVA. Symbols: ++++p<0.0001 vs baseline (BL), °°p<0.01, °°°p<0.001 vs respective Sham controls. Abbreviation: M=males, F=females.

**Supplementary Figure 3**

**Suppl. Figure 3. Endocannabinoid and *N*-acylethanolamine levels in pain-related brain areas and lumbar spinal cord in male and female MIA or Sham rats.** Endocannabinoids (2-AG, AEA) and *N*-acylethanolamines (PEA, OEA) levels in: (**a-d**) periaqueductal grey (PAG), (**e-h**) rostroventral medulla (RVM), (**i-l**) prefrontal cortex (PFC), (**m-p**) thalamus (THA), and (**q-t**) dorsal lumbar (L3-L6) spinal cord of male and female MIA or Sham rats. Data are expressed as mean ± SEM (n=3-9 per group) and analysed using two-way (**a-p**) or three-way ANOVA **(q-t)** (or corresponding mixed-effects model due to outlier removal). Abbreviation: M=males, F=females.
